# Pulsed radiofrequency of dorsal root ganglion of upper thoracic segment for herpes zoster neuralgia: Case report

**DOI:** 10.1097/MD.0000000000020807

**Published:** 2020-06-19

**Authors:** Yong Fei, Jiajia Deng, Hui Lv, Ming Yao, Tingting Wang, Bing Huang

**Affiliations:** Department of Anesthesiology and Pain Medicine, The Affiliated Hospital of Jiaxing University, Jiaxing, Zhejiang, China.

**Keywords:** dorsal root ganglion, pulsed radiofrequency, thoracic sympathetic nerve

## Abstract

**Rationale:**

Pulsed radiofrequency (PRF) therapy of dorsal root ganglion is effective in treating acute stage shingles neuralgia of chest and back. Herein, a case of herpetic neuralgia with difficult puncture of dorsal root ganglion of upper thoracic segment is report.

**Patient concerns:**

A 62-year-old male patient was admitted to the hospital for 2 days for herpes zoster with paroxysmal needle-like pain in the left chest and back. The skin lesion area of herpes zoster and the superficial location of neuralgia was left T2–4, and visual analog scale (VAS) score was 6 points.

**Diagnosis:**

Two days ago, the patient had paroxysmal needle-like pain in the left chest and back, without herpes, and was admitted to the hospital for emergency treatment. Chest pain and myocardial infarction were considered; however, troponin, myocardial enzyme spectrum, and blood amylase were in the normal range. On the evening of the same day, the patient presented green bean-sized blisters distributed in clusters along the left T2–4 nerve as a banded pattern. Thus, the patient was diagnosed as shingles.

**Intervention:**

Oral gabapentin capsules, varaciclovir tablets, mecobalamine tablets, and amitriptyline hydrochloride tablets were administered, and topical aciclovir cream was applied. The VAS score after the above treatment was 5 points. The patient underwent computed tomography-guided PRF surgery on the dorsal root ganglion.

**Outcome:**

Postoperative pain was relieved. One month post-surgery, no oral analgesic drugs were administered. The VAS score was 1 point, and the pain completely disappeared at 3 months post-surgery.

**Conclusions:**

Herpes zoster is most common in the chest and back. The PRF of dorsal root ganglion cannot access the target by conventional puncture, and can be completed by thoracic sympathetic nerve radiofrequency puncture path.

## 1 Introduction

Herpes zoster is caused by varicella-herpes zoster virus infection of the dorsal root ganglion or trigeminal semilunar ganglion. It is more common in middle-aged and elderly individuals with reduced immunity, among whom the proportion of patients with shingles in the chest and back is maximal.^[1,2]^ Pulse radiofrequency (PRF) of dorsal root ganglion is effective in treating shingles neuralgia in the chest and back, which greatly reduces the incidence of the infection.^[3,4]^

The biggest difficulty in PRF of dorsal root ganglion of upper thoracic segment is puncture of the target. In clinical cases, the puncture path is often blocked by ribs or transverse processes, rendering it difficult to puncture to the target position (the upper quadrant of the ventral foramen), resulting in surgical failure or reduced effect.

In this study, computed tomography (CT)-guided thoracic sympathetic nerve radiofrequency technique (T4) was used to treat Raynaud syndrome or anhidrosis, and a good therapeutic effect was achieved.^[5]^ In this case, the puncture route was adjusted according to the thoracic sympathetic nerve puncture to reach the target smoothly.

## 2 Case report

A 62-year-old male was admitted to the hospital for emergency treatment for 2 days for herpes zoster with paroxysmal needle-like pain in the left chest and back, without herpes, and went to the hospital. Chest pain and myocardial infarction were considered. The level of troponin, myocardial enzyme spectrum, and blood amylase was normal. On the evening of the same day, the patient presented a green bean-sized blister distributed in clusters along the left T2–4 nerve, presenting a banded pattern, which led to the diagnosis of shingles in the patient. After admission, the patient received oral gabapentin capsules 0.3 Po tid, valaciclovir tablets 0.3 Po bid, mecobalamine tablets 0.5 mg Po tid, and amittiline hydrochloride tablets 12.5 mg Po qn, and topical aciclovir cream was applied 4 times a day. The visual analog score score after the above treatment was 5 points. Informed consent was obtained from the patient to publish the case, and approval for this study was obtained from the Medical Ethics Committee of the First Hospital of Jiaxing (No. LS2018-240).

**Figure 1:**
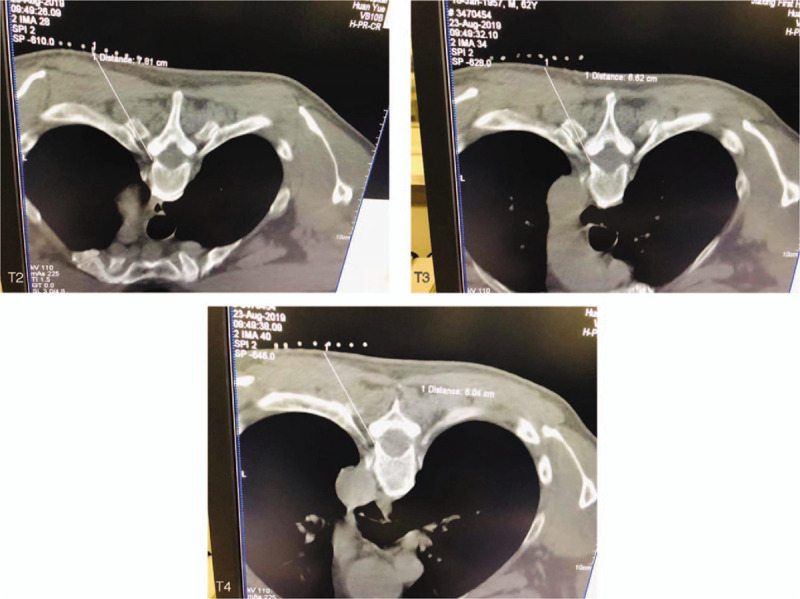
The left T2, T3, and T4 dorsal root ganglion puncture routes were designed by CT scan. CT = computed tomography.

**Figure 2:**
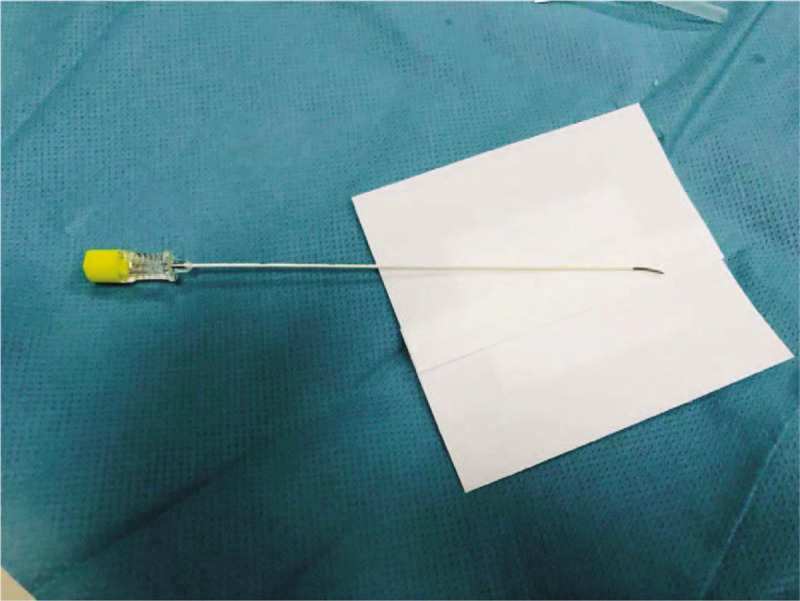
The exposed end of the 20 G puncture needle with a length of 150 mm and the movable end with a length of 10 mm at a specific angle; it was ensured that the needle core could be pulled out.

**Figure 3:**
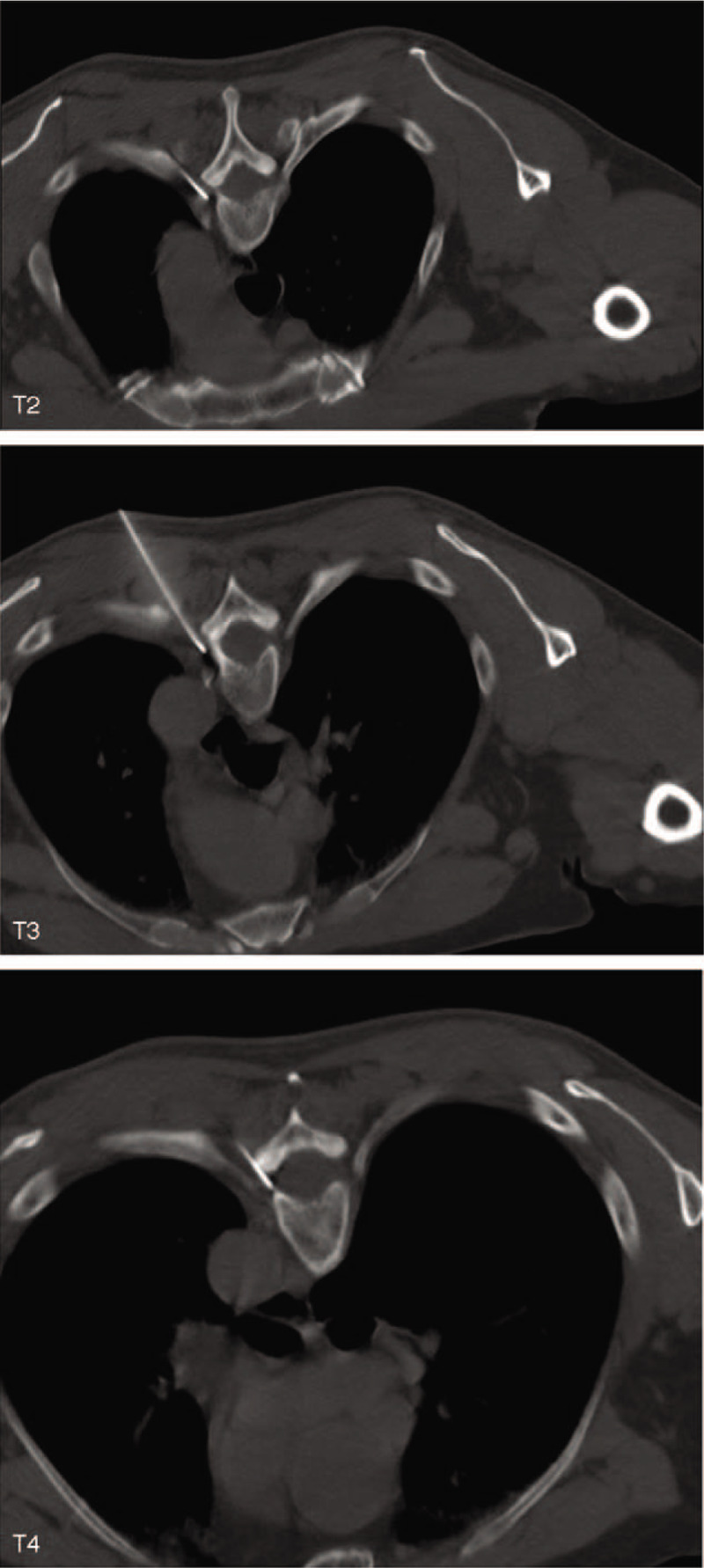
CT-guided puncture of left T2, T3, and T4 dorsal root ganglion to 1/3 of the intervertebral foramen. CT = computed tomography.

The patient underwent CT-guided PRF surgery on the dorsal root ganglion. The patient lay in the prone position on the CT treatment bed, with a severe pain segment as the center (left T3), and the upper and lower segments were extended by 1 each. The upper edge of the ventral intervertebral foramen was selected as the puncture point by CT positioning to decide the puncture path (Fig. 1). Consequently, the puncture path was found to be blocked by ribs or transverse processes, making it difficult to puncture to the target position. Based on the application of the curved needle technology in the radiofrequency therapy of trigeminal neuralgia and Raynaud syndrome,^[5–7]^ we bent the exposed end of the puncture needle of the same material (20 G, 150 mm in length and 10 mm in length at the moving end) to a certain angle and ensured that the needle core could be pulled out (Fig. 2). The skin puncture point was moved up by 2 to 2.5 cm according to the design of the puncture path, such that the whole puncture needle enters at a specific angle from the skin puncture to reach the target to complete the operation. After the puncture path design, regular disinfection, towels, 1.0% lidocaine hydrochloride local anesthetic infiltration, and needle-induced broken skin, the pricked elbow points were exposed to ensure that it is easy to puncture needle to rib end transverse joint. The CT scan was performed after the needle bared the cutting-edge puncture transverse process to the rib joint, the needle was moved slightly back to the puncture point, rotated 180°, and slowly advanced. The CT scan confirmed that the needle crosses the cross rib joint, and then, it was rotated again 180°, exposed with the end pointing in the direction of the intervertebral foramen, and fine-tuned to reach the target position (Fig. 3). The radiofrequency instrument (Baylis Medical Inc., Montreal, Canada) was utilized for a sensory test at the following parameters: voltage: 0.1 to 0.3 V, frequency: 50 Hz; this induced discomfort such as acid, swelling, numbness or tingling in the original pain area. Low-frequency current and setting parameters were adopted in the exercise test. The voltage was 0.4 to 1.0 V, the frequency was 2 Hz, and no corresponding trunk muscle fibrillation and pulsation were observed in the corresponding segments. The temperature, time, pulse width, and frequency were set at 42°C, 360 seconds, 20 ms, and 2 Hz, respectively. After PRF, the needle electrode core was removed, the needle was withdrawn with no blood, gas or liquid, and each section was injected with 5 mL fluid. Typically, for 3 sections of treatment, the fluid consisting of 2% lidocaine hydrochloride 100 mg, methyl cobalamin injection 1 mg, tamethasone 1 mg, recombinant human interferon alpha-2b 1 million U, and 30% iodinitol 3 mL was added into 0.9% normal saline in a 15 mL reaction volume. After the needle is removed, the puncture point was compressed. After 15-min observation, the patient regained normal vital signs and was allowed to return to the ward.

Postoperative pain was relieved. At 1 month after the surgery, no oral analgesic drugs were administered. The visual analog scale score of the patient was 1 point, and the pain completely disappeared at 3 months post-surgery.

## 3 Discussion

PRF uses pulsed current to restrict the heat generation at <42°, resulting in tissue damage of the dorsal root ganglion at the lowest level of ultrastructure.^[8,9]^ Recent studies have shown that Na/K ATPase and c-fos expression is upregulated in the spinal cord after PRF therapy^[10]^ with increasing changes in the synaptic transmission.^[11]^ These mechanisms might lead to changes in neuroplasticity that enhance the therapeutic effect of PRF. Therefore, PRF is widely used in the clinical treatment of herpes zoster neuralgia.

**Figure 4:**
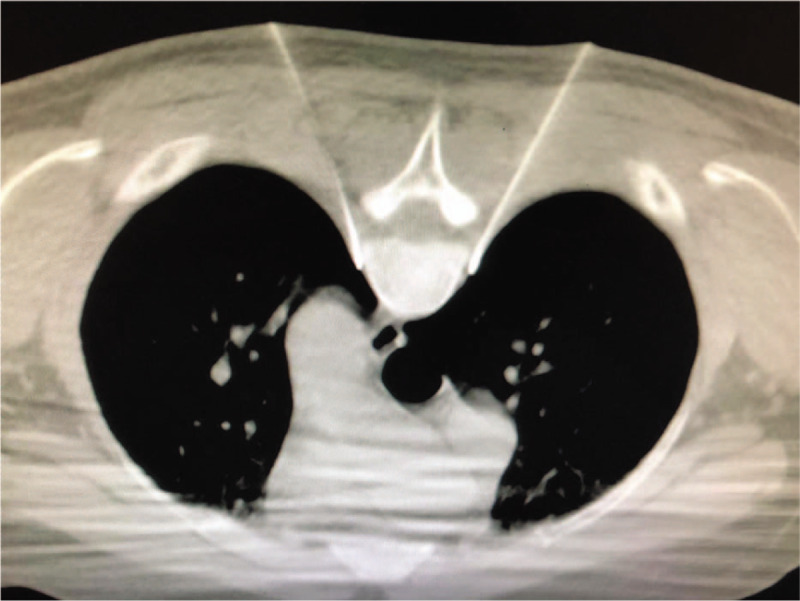
The bilateral T4 sympathetic radiofrequency needle penetrates the lateral side of the rib and is attached to the parietal pleura.

CT-guided thoracic sympathetic nerve radiofrequency technique was used to treat Raynaud syndrome or anhidrosis, and the puncture target was achieved successfully.^[5]^ The present study reported that the curved needle puncture technique could be applied to the chest sympathetic nerve radiofrequency for the treatment of Raynaud syndrome or hyperhidrosis for an improved therapeutic effect. The steps were as follows: the exposed end of the puncture needle (20 G, 100 mm in length, and the tip of the blunt needle with a moving end length of 10 mm) was bent to a certain angle and ensured that the needle core could be pulled out. The skin puncture point was moved up by 2 to 2.5 cm according to the design of the puncture path, such that the whole puncture needle enters at a specific angle from the skin puncture to reach the target to complete the operation. After the puncture path design, regular disinfection, towels, 1.0% lidocaine hydrochloride local anesthetic infiltration, and needle-induced broken skin, the pricked elbow points were exposed to ensure that it is easy to puncture needle to rib end transverse joint. The CT scan was performed after the needle bared the cutting-edge puncture transverse process to the rib joint, the needle was moved slightly back to the puncture point, rotated 180°, and slowly advanced. The CT scan confirmed that the needle crosses the cross rib joint, and then, it was rotated again 180°, exposed with the end pointing in the direction of the intervertebral foramen. The CT-guided puncture needle clings to the small head of the ribs and pleural and moved forward slowly. The tip of the final puncture needle is located lateral to the rib and close to the parietal pleura (Fig. 4). Inspired by the chest sympathetic radiofrequency puncture path and the needle bending technique, the exposed end of the needle was bent at a specific angle to ensure that the needle core could be pulled out. The whole puncture process was similar to chest sympathetic radiofrequency puncture, except that after the puncture needle passes the costal transverse facet joint, the direction of the puncture needle needs to be adjusted in the direction of 1/3rd of the intervertebral foramen to complete the operation. For difficult upper thoracic dorsal root ganglion puncture, this method is suggested to be successful.

## 4 Conclusions

In conclusion, the PRF of dorsal root ganglion is a common method in the treatment of herpes zoster in the chest and back. For the difficult puncture of the dorsal root ganglion, PRF can be achieved by the thoracic sympathetic nerve radio frequency puncture path.

## Author contributions

YF conducted the majority of the experiments. JJD wrote the manuscript. HL conceived and designed the study. MY performed the data analysis and collected the data. BH reviewed the final draft and TTW coordinated and supervised the experiments. All authors read and approved the final manuscript.
